# Dataset, including a photo-guide, of alien plants sold in traditional medicine markets and healthcare outlets in three South African cities, specifically by traders of Indian, West African, East African, and Chinese origin

**DOI:** 10.1016/j.dib.2021.107395

**Published:** 2021-09-21

**Authors:** Vivienne L. Williams, Amy Burness, Ewa M. Wojtasik, Marcus J. Byrne

**Affiliations:** a School of Animal, Plant and Environmental Sciences, University of the Witwatersrand, Johannesburg Wits 2050, South Africa; b Pretoria National Botanical Garden, South African National Biodiversity Institute, Pretoria, South Africa; c DSI-NRF Centre of Excellence for Invasion Biology, School of Animal, Plant and Environmental Sciences, University of the Witwatersrand, Johannesburg Wits 2050, South Africa

**Keywords:** African traditional medicine, Alien plants, Ethnomedicine, Inventory, Urban migrant ethnobotany

## Abstract

This dataset is a an inventory of 475 alien plant taxa (447 identified to species), including a photo-guide to 96 plants, mostly sold as traditional medicines in three South African cities by traders of South African, West African, East African, Indian and Chinese origin (Williams et al., 2021). The dataset also incorporates species documented in a literature survey of alien plants used for traditional medicines in South Africa. The species inventory is a consolidation of the data from two separate investigations of 106 medicinal plant traders: firstly, a study conducted in 2010/2011 of 77 traders in markets and shops in Johannesburg, Pretoria and Durban (Williams et al., 2021); and secondly, a study conducted in 2017/2018 of plants sold by 29 (im)migrant traders of West African, East African, and Indian origin in Johannesburg, and of alien species listed in a TCM (Traditional Chinese Medicine) catalogue (Burness, 2019). Accompanying each plant photograph in the photo-guide is the following information: species name; common name(s) provided by the survey respondents; invasive alien plant category; introduction status; voucher specimen number(s); nationality of the medicine traders; and, notes on source localities (e.g. imported or collected in southern Africa). Overall, most of the taxa were from the Asteraceae (12%), Fabaceae (9%) and Poaceae (5%). The species are mostly unlisted (76%) with respect to their legal status in South Africa in terms of the National Environmental Management: Biodiversity Act (NEM:BA), 2004 Alien & Invasive Species (A&IS) regulations. The most frequently recorded species in the various surveys were *Glycyrrhiza glabra, Acorus calamus, Angelica sinensis* and *Zingiber officinale.*

## Specifications Table

**Table:** 

Subject	Ethnobotany, Ethnomedicine
Specific subject area	Medicinal plants; alien species
Type of data	Table Image Graph
How data were acquired	Fieldwork investigations of traditional medicine markets, healthcare outlets and shops; herbarium data; bibliographic research; literature survey
Data format	Raw Analyzed
Parameters for data collection	Alien plants identified in traditional healthcare outlets and markets, as well as imported species sold by traders of Indian, West African, East African, and Chinese origin.
Description of data collection	Fieldwork through visits to traditional medicine markets, healthcare outlets and shops where plants were inventoried, identified and classified. Also, bibliographic research and a literature survey. Photographs were taken of purchased specimens to create a reference library.
Data source location	Cities: Johannesburg, Pretoria, Durban Country: South Africa
Data accessibility	With the article
Related research article	V.L. Williams, E.M. Wojtasik, M.J. Byrne, 2021, A chronicle of alien medicinal plants used as traditional medicine in South Africa, with their status as invasive weeds, S. Afr. J. Bot. 142, 63–72. https://doi.org/10.1016/j.sajb.2021.05.027

## Value of the Data

• The dataset records a consolidated list of 475 identified alien plant taxa (including photographs for 96) used as traditional medicines in South Africa (some of which are imported), and sold in a range of traditional medicine outlets.
• The photographs and common names serve as a guide/reference library to assist researchers with identifying alien plants recorded in surveys of traditional medicine markets.
• The data are relevant to researchers investigating traditional medicine use, urban migrant ethnobotany, and invasion biology.
• This inventory will provide a basis for future legislation in relation to regulation of alien plant species control.

## 1 Data Description

The data are presented as: (i) photographs taken of alien plants sold in Johannesburg, Pretoria and Durban in 2010/2011, as well as of plants sold by West and East African and Indian immigrants in Johannesburg 2017/2018 (Tables 1 and 2, for identified and unidentified plants respectively); and (ii) a consolidated inventory of the alien plants used for traditional medicine (TM, or *muthi*) in South Africa, some of which are imported from the home countries of the TM traders of West and East African, Indian and Chinese origin (Table 3).

**Table 1:** Photographic guide to identified alien plants sold for traditional medicine by traders in three South Africa cities. The key to the data in the tables underneath the photographs is in Fig. 1. The same scale bar is used in each image.

**Table 2:** Photographic guide to the unidentified alien plants sold for traditional medicine by traders in three South Africa cities. The key to the data in the tables underneath the photographs is in Fig. 1.

**Table 3:** Inventory of 475 plant, fungi and algae taxa inventoried in traditional medicine outlets and markets in three South African cities, a TCM catalogue, and from the literature. The data are a consolidation of two studies conducted in 2010/2011 [1] and 2017/2018 [3]. *Information source* refers to the year of the study and the origin of the information, where: *A* = 2010/2011; *B* = 2017/2018; *market* = obtained from a survey of traders selling alien medicinal plants in markets or outlets such as shops in Johannesburg, Pretoria and/or Durban; *in lit.* = obtained from a literature survey of alien plants used in South Africa; *market & in lit.* = recorded in the literature as being sold in a market/outlet in South Africa; *TCM-C* = obtained from a catalogue of Traditional Chinese Medicine remedies sold in at least four franchises in Johannesburg. The *legal invasive status* (the species’ status in terms of NEM:BA A&IS regulations, from [4], [5], [6]), where: category 1a = invasive species that must be combatted or nationally eradicated; 1b = invasive species that must be controlled in accordance with a national management programme, and cannot be traded or otherwise allowed to spread; 2 = invasive species that are the same as category 1b species except that permits are required to carry out restricted activities (cultivation, ownership and trade), and they must be controlled in the absence of a permit. Persons in control of these species, or persons in possession of permits, must ensure that specimens of the species do not spread outside the areas specified on the permits; 3 = invasive species that are subject to exemptions and can be kept without permits, but they cannot be further traded or propagated and must be controlled. Species occurring in riparian zones must be treated as category 1b species and managed accordingly; unlisted = alien species that are not listed in the regulations, but that have been reported as present outside of captivity or cultivation in South Africa or on offshore islands. *Introduction status* in South Africa (from [7,8]), where: A0 = never introduced beyond limits of indigenous range to South Africa; C0 = individuals released outside of captivity or cultivation in location where introduced, but incapable of surviving for a significant period; C2 = individuals surviving outside of captivity or cultivation in location where introduced, but population not self-sustaining; D1 = self-sustaining population outside of captivity or cultivation, with individuals surviving a significant distance from the original point of introduction; E = fully invasive species, with individuals dispersing, surviving and reproducing at multiple sites across a greater or less spectrum of habitats and extent of occurrence; introduced = introduced, but no other data available. *Outlet and/or TCM catalogue frequency* indicates the numbers of records for species that were sold in different traditional medicine outlets, and/or recorded in the TCM catalogue. Research conducted in 2011 (A) was with South African traders selling alien plants (including traders of Indian descent). Research conducted in 2017/2018 (B) was with post-1994 (im)migrant traders of West African, East African, Indian and Chinese origin. Blank cells indicate species that were not recorded during our market surveys. *CITES listing:* Eight CITES listed species are indicated as bold superscripts next to the species, with CAI = CITES Appendix I, and CAII = CITES Appendix II; the CITES listing for *Panax ginseng* applies only to the population of the Russian Federation.

					Outlet and/or TCM catalogue frequency		
Species	Family	Information source	Legal invasive status category	Introduction status	2011 markets	2017 markets	TCM-C
*Abelmoschus esculentus* (L.) Moench	Malvaceae	*B (2017 market)*	Unlisted			4	
*Acacia decurrens* (J.C.Wendl.) Willd.	Fabaceae	*A (in lit.)*	2	E			
*Acacia farnesiana* (L.) Willd.	Fabaceae	*A (in lit.)*	Unlisted				
*Acacia melanoxylon* R.Br.	Fabaceae	*A (in lit.)*	2	E			
*Acacia pycnantha* Benth.	Fabaceae	*A (in lit.)*	1b	E			
*Acanthospermum australe* (Loefl.) Kuntze	Asteraceae	*A (in lit.)*	Unlisted	C2			
*Acanthospermum glabratum* (DC.) Wild	Asteraceae	*A (in lit.)*	Unlisted				
*Acanthospermum hispidum* DC.	Asteraceae	*A (market & in lit.)*	Unlisted	E			
*Acanthus polystachyus* Delile	Acanthaceae	*A (in lit.)*	Unlisted				
*Achillea millefolium* L.	Asteraceae	*A (in lit.)*	Unlisted	E			
*Achyranthes aspera* L. var. aspera	Amaranthaceae	*A (in lit.)*	Unlisted	E			
*Achyranthes bidentata* Blume	Amaranthaceae	*B (2017 TCM-C)*	Unlisted				5
*Acmella caulirhiza* Delile	Asteraceae	*A (2011 market)*	Unlisted		5		
*Aconitum fischeri* Rchb.	Ranunculaceae	*B (2017 TCM-C)*	Unlisted				3
*Acorus calamus* L.	Acoraceae	*A (2011 market); B (2017 TCM-C)*	Unlisted	E	31		1
*Acridocarpus* sp.	Malpighiaceae	*B (2017 market)*				4	
*Actaea dahurica* (Turcz. ex Fisch. & C.A.Mey.) Franch.	Ranunculaceae	*B (2017 TCM-C)*	Unlisted				1
*Aframomum* cf. *corrorima* (A.Braun) P.C.M.Jansen	Zingiberaceae	*B (2017 market)*	Unlisted			4	
*Aframomum melegueta* K.Schum.	Zingiberaceae	*B (2017 market)*	Unlisted			3	
*Afrohybanthus enneaspermus* (L.) Flicker	Violaceae	*A (in lit.)*	Unlisted				
*Agave americana* L. subsp. *americana* var. *americana*	Agavaceae	*A (in lit.)*	Unlisted	E			
*Agave sisalana* Perrine	Agavaceae	*A (market & in lit.)*	2	E			
*Agave* sp.	Agavaceae	*A (in lit.)*					
*Ageratum conyzoides* L.	Asphodelaceae	*A (in lit.)*	1b	E			
*Ailanthus altissima* (Mill.) Swingle	Simaroubaceae	*A (in lit.)*	1b	E			
*Aira cupaniana* Guss.	Poaceae	*A (in lit.)*	Unlisted				
*Albizia julibrissin* Durazz.	Fabaceae	*B (2017 TCM-C)*	Unlisted				1
*Albizia lebbeck* (L.) Benth.	Fabaceae	*A (in lit.)*	1b	E			
*Alisma plantago-aquatica* L.	Alismataceae	*B (2017 TCM-C)*	1b	E			13
*Allium cepa* L.	Amaryllidaceae	*A (in lit.)*	Unlisted				
*Allium sativum* L.	Amaryllidaceae	*A (in lit.); B (2017 market)*	Unlisted			3	
*Allium tuberosum* Rottler ex Spreng.	Amaryllidaceae	*B (2017 TCM-C)*	Unlisted				1
*Alpinia officinarum* Hance	Zingiberaceae	*B (2017 TCM-C)*	Unlisted				1
*Alternanthera sessilis* (L.) R.Br. ex DC.	Amaranthaceae	*A (in lit.)*	Unlisted				
*Amaranthus caudatus* L.	Amaranthaceae	*A (in lit.)*	Unlisted				
*Amaranthus cruentus* (L.)	Amaranthaceae	*A (in lit.)*	Unlisted	E			
*Amaranthus spinosus* L.	Amaranthaceae	*A (in lit.)*	Unlisted				
*Amomum maximum* Roxb.	Zingiberaceae	*B (2017 TCM-C)*	Unlisted				1
*Amomum subulatum* Roxb.	Zingiberaceae	*B (2017 market)*	Unlisted			4	
*Amorphophallus paeoniifolius* (Dennst.) Nicolson	Araceae	*B (2017 market)*	Unlisted			1	
*Anacardium occidentale* L.	Anacardiaceae	*A (2011 market)*	Unlisted		2		
*Anagallis arvensis* L. subsp. arvensis	Primulaceae	*A (in lit.)*	Unlisted	C2			
*Andrographis paniculata* (Burm.f.) ex Nees	Acanthaceae	*A (2011 market)*	Unlisted		1		
*Anemarrhena asphodeloides* Bunge	Asparagaceae	*B (2017 TCM-C)*	Unlisted				3
*Anethum graveolens* L.	Apiaceae	*A (in lit.); B (2017 market); B (2017 TCM-C)*	Unlisted			3	1
*Angelica dahurica* (Hoffm.) Benth. & Hook.f. ex Franch. & Sav.	Apiaceae	*B (2017 TCM-C)*	Unlisted				5
*Angelica pubescens* Maxim.	Apiaceae	*B (2017 TCM-C)*	Unlisted				1
*Angelica sinensis* (Oliv.) Diels	Apiaceae	*B (2017 TCM-C)*	Unlisted				30
*Annickia chlorantha* (Oliv.) Setten & Maas	Annonaceae	*B (2017 market)*	Unlisted			7	
*Annona reticulata* L.	Annonaceae	*A (market & in lit.); B (2017 market)*	Unlisted			1	
*Anredera cordifolia* (Ten.) Steenis	Basellaceae	*A (2011 market)*	1b	E	8		
*Anthemis cotula* L.	Asteraceae	*A (in lit.)*	Unlisted	C2			
*Anthoxanthum odoratum* L. var. *odoratum*	Poaceae	*A (in lit.)*	Unlisted				
*Anthriscus sylvestris* var. *sylvestris* (L.) Hoffm.	Apiaceae	*A (in lit.)*	Unlisted				
*Apium graveolens* L.	Apiaceae	*A (in lit.)*	Unlisted	C2			
*Apocynum venetum* L	Apocynaceae	*B (2017 TCM-C)*	Unlisted				1
*Arachis hypogaea* L.	Fabaceae	*A (in lit.)*	Unlisted				
*Araucaria bidwillii* Hook.	Araucariaceae	*A (in lit.)*	Unlisted	E			
*Araucaria heterophylla* (Salisb.) Franco	Araucariaceae	*A (2011 market)*	Unlisted		4		
*Araujia sericifera* Brot.	Apocynaceae	*A (in lit.)*	1b	E			
*Arctium lappa* L.	Asteraceae	*B (2017 TCM-C)*	Unlisted				1
*Areca catechu* L.	Arecaceae	*A (2011 market); B (2017 TCM-C)*	Unlisted		2		1
*Argemone mexicana* L.	Papaveraceae	*A (in lit.)*	1b	E			
*Argemone ochroleuca* Sweet	Papaveraceae	*A (in lit.)*	1b	E			
*Artemisia absinthium* L.	Asteraceae	*A (in lit.)*	Unlisted				
*Artemisia annua* L.	Asteraceae	*B (2017 market)*	Unlisted			1	
*Artemisia scoparia* Waldst. & Kitam.	Asteraceae	*B (2017 TCM-C)*	Unlisted				1
*Arundo donax* L.	Poaceae	*A (in lit.)*	1b	E			
*Asarum heterotropoides* F.Schmidt	Aristolochiaceae	*B (2017 TCM-C)*	Unlisted				4
*Asimina* sp.	Annonaceae	*B (2017 market)*				1	
*Asparagus cochinchinensis* (Lour.) Merr.	Asparagaceae	*B (2017 TCM-C)*	Unlisted				1
*Astragalus mongholicus* Bunge	Fabaceae	*B (2017 TCM-C)*	Unlisted				14
*Atractylodes lancea* (Thunb.) DC.	Asteraceae	*B (2017 TCM-C)*	Unlisted				2
*Atractylodes macrocephala* Koidz.	Asteraceae	*B (2017 TCM-C)*	Unlisted				14
*Atriplex nummularia* Lindl.	Chenopodiaceae	*A (in lit.)*	2	E			
*Aucklandia costus* Falc. **^CAI^**	Asteraceae	*B (2017 TCM-C)*	Unlisted				1
*Avena fatua* L.	Poaceae	*A (in lit.)*	Unlisted	E			
*Avena sativa* L.	Poaceae	*A (in lit.)*	Unlisted	C0			
*Azadirachta indica* A.Juss.	Meliaceae	*B (2017 market)*	Unlisted			3	
*Bauhinia variegata* L.	Fabaceae	*A (in lit.)*	1b EC, KZN, LP, MP; 3 elsewhere	E			
*Beta vulgaris* L. subsp. *vulgaris*	Chenopodiaceae	*A (in lit.)*	Unlisted				
*Bidens bipinnata* L.	Asteraceae	*A (in lit.)*	Unlisted	E			
*Bidens pilosa* L.	Asteraceae	*A (in lit.)*	Unlisted	E			
*Bletilla striata* (Thunb.) Rchb.f. **^CAII^**	Orchidaceae	*B (2017 TCM-C)*	Unlisted				1
*Boswellia* sp.	Burseraceae	*B (2017 market)*				3	
*Brassica juncea* (L.) Czern.	Brassicaceae	*A (in lit.)*	Unlisted				
*Brassica nigra* (L.) W.D.J.Koch	Brassicaceae	*A (in lit.)*	Unlisted				
*Brassica rapa* L.	Brassicaceae	*A (in lit.)*	Unlisted	C2			
*Brassica* sp.	Brassicaceae	*A (2011 market); B (2017 market)*			3	3	
*Bromus catharticus* Vahl.	Poaceae	*A (in lit.)*	Unlisted	E			
*Bryophyllum* sp.	Crassulaceae	*A (2011 market)*			4		
*Buglossoides arvensis* (L.) I.M.Johnst.	Boraginaceae	*A (in lit.)*	Unlisted				
*Bunium bulbocastanum* L.	Apiaceae	*B (2017 market)*	Unlisted			1	
*Bupleurum chinense* DC.	Apiaceae	*B (2017 TCM-C)*	Unlisted				16
*Caesalpinia pulcherrima* (L.) Sw.	Fabaceae	*A (in lit.)*	Unlisted	C2			
*Cannabis sativa* L.	Cannabaceae	*A (in lit.)*	Unlisted				
*Capsella bursa-pastoris* (L.) Medik.	Brassicaceae	*A (in lit.)*	Unlisted	C2			
*Cardiospermum halicacabum* L.	Sapindaceae	*A (in lit.)*	3	E			
*Carduus tenuiflorus* Curtis	Asteraceae	*A (in lit.)*	Unlisted				
*Carica papaya* L.	Caricaceae	*A (in lit.)*	Unlisted	E			
*Carthamus lanatus* L.	Asteraceae	*A (in lit.)*	Unlisted				
*Carthamus tinctorius* L.	Asteraceae	*B (2017 TCM-C)*	Unlisted				6
*Cassia fistula* L.	Fabaceae	*A (2011 market)*	Unlisted		3		
*Cassytha filiformis* L.	Lauraceae	*A (market & in lit.)*	Unlisted				
*Casuarina equisetifolia* L.	Casuarinaceae	*A (in lit.)*	2	E			
*Catharanthus roseus* (L.) G.Don	Apocynaceae	*A (in lit.)*	1b excluding sterile cultivars	E			
*Cenchrus americanus* (L.) Morrone	Poaceae	*A (in lit.)*	Unlisted	introduced			
*Cenchrus clandestinus* (Hochst. ex Chiov.) Morrone	Poaceae	*A (in lit.)*	1b (see NEM:BA)	E			
*Cenchrus purpureus* (Schumach.) Morrone	Poaceae	*A (in lit.)*	2	E			
*Centaurea benedicta* (L.) L.	Asteraceae	*A (in lit.)*	Unlisted				
*Centaurea calcitrapa* L.	Asteraceae	*A (in lit.)*	Unlisted				
*Centaurea cyanus* L.	Asteraceae	*A (in lit.)*	Unlisted				
*Centaurea melitensis* L.	Asteraceae	*A (in lit.)*	Unlisted	C2			
*Centaurea solstitialis* L.	Asteraceae	*A (in lit.)*	Unlisted	C2			
*Centella asiatica* (L.) Urb.	Apiaceae	*A (in lit.)*	Unlisted				
*Ceratonia siliqua* L.	Fabaceae	*A (in lit.)*	Unlisted				
*Cereus jamacaru* DC.	Cactaceae	*A (market & in lit.)*	1b	E			
*Cestrum laevigatum* Schltdl.	Solanaceae	*A (in lit.)*	1b	E			
*Chenopodium album* L.	Chenopodiaceae	*A (in lit.)*	Unlisted	E			
*Chenopodium opulifolium* Schrad. ex W.D.J.Koch & Ziz	Chenopodiaceae	*A (in lit.)*	Unlisted				
*Chrysanthemum indicum* L.	Asteraceae	*B (2017 TCM-C)*	Unlisted				1
*Chrysanthemum morifolium* Ramat. Hemsl.	Asteraceae	*B (2017 TCM-C)*	Unlisted				5
*Chrysopogon zizanioides* (L.) Roberty	Poaceae	*A (in lit.)*	Unlisted				
*Cicer arietinum* L.	Fabaceae	*B (2017 market)*	Unlisted			3	
*Cichorium intybus* L. subsp. *intybus*	Asteraceae	*A (in lit.)*	Unlisted	E			
*Cinnamomum camphora* (L.) J. Presl.	Lauraceae	*A (2011 market)*	1b EC, KZN, LP, MP and 3 WC	E			
*Cinnamomum* sp.	Lauraceae	*A (2011 market)*			3		
*Cinnamomum verum* J.Presl	Lauraceae	*A (market & in lit.); B (2017 market); B (2017 TCM-C)*	Unlisted		7	7	1
*Cirsium arvense* (L.) Scop.	Asteraceae	*A (in lit.)*	Unlisted	E			
*Cirsium vulgare* (Savi) Ten.	Asteraceae	*A (market & in lit.)*	1b	E			
*Cistanche deserticola* Y.C.Ma **^CAII^**	Orobanchaceae	*B (2017 TCM-C)*	Unlisted				1
*Citrus limon* (L.) Burm.f.	Rutaceae	*A (in lit.)*	Unlisted	E			
*Citrus* x *aurantium* L.	Rutaceae	*B (2017 TCM-C)*	Unlisted				8
*Clematis armandii* Franch.	Ranunculaceae	*B (2017 TCM-C)*	Unlisted				1
*Clematis* sp.	Ranunculaceae	*B (2017 TCM-C)*					3
*Cnidium monnieri* (L.) Cusson	Apiaceae	*B (2017 TCM-C)*	Unlisted				1
*Cocculus hirsutus* (L.) W.Theob.	Menispermaceae	*A (in lit.)*	Unlisted				
*Coffea* sp.	Rubiaceae	*B (2017 market)*				2	
*Coix lacryma-jobi* L.	Poaceae	*A (2011 market); B (2017 TCM-C)*	Unlisted	E	9		2
*Cola acuminata* (P.Beauv.) Schott & Endl.	Malvaceae	*B (2017 market)*	Unlisted			5	
*Colocasia esculenta* (L.) Schott	Araceae	*A (market & in lit.)*	Unlisted	E			
*Commelina benghalensis* L.	Commelinaceae	*A (2011 market)*	Unlisted		1		
*Commiphora* sp.	Burseraceae	*B (2017 TCM-C)*					1
*Convolvulus arvensis* L.	Convolvulaceae	*A (in lit.)*	1b	E			
*Coptis chinensis* Franch.	Ranunculaceae	*B (2017 TCM-C)*	Unlisted				10
*Coriandrum sativum* L.	Apiaceae	*A (2011 market); B (2017 market)*	Unlisted		2	3	
*Cornus officinalis* Siebold & Zucc.	Cornaceae	*B (2017 TCM-C)*	Unlisted				7
*Corydalis yanhusuo* (Y.H.Chou & Chun C.Hsu) W.T.Wang ex Z.Y.Su & C.Y.Wu	Papaveraceae	*B (2017 TCM-C)*	Unlisted				2
*Corymbia ficifolia* (F.Muell.) K.D.Hill & L.A.S.Johnson	Myrtaceae	*A (in lit.)*	Unlisted	E			
*Cosmos bipinnatus* Cav.	Asteraceae	*A (in lit.)*	Unlisted	E			
*Crataegus laevigata* (Poir.) DC.	Rosaceae	*B (2017 TCM-C)*	Unlisted				1
*Crataegus pinnatifida* Bunge	Rosaceae	*B (2017 TCM-C)*	Unlisted				2
*Crinitaria tatarica* (Less.) Soják	Asteraceae	*B (2017 TCM-C)*	Unlisted				2
*Crotalaria brevidens* var. *intermedia* (Kotschy) Polhill	Fabaceae	*A (in lit.)*	Unlisted				
*Crotalaria juncea* L.	Fabaceae	*A (in lit.)*	Unlisted				
*Cucumis melo* L.	Curcurbitaceae	*B (2017 market)*	Unlisted			1	
*Cuminum cyminum* L.	Apiaceae	*B (2017 market)*	Unlisted			4	
*Cupressus* sp.	Cupressaceae	*A (market & in lit.)*					
*Curculigo orchioides* Gaertn.	Hypoxidaceae	*B (2017 TCM-C)*	Unlisted				1
*Curcuma longa* L.	Zingiberaceae	*A (2011 market); B (2017 market)*	Unlisted		5	7	
*Curcuma* sp.	Zingiberaceae	*B (2017 TCM-C)*					1
*Cuscuta chinensis* Lam.	Convolvulaceae	*B (2017 TCM-C)*	Unlisted				1
*Cuscuta epithymum* (L.) L.	Convolvulaceae	*A (in lit.)*	Unlisted				
*Cyathula officinalis* K.C.Kuan	Amaranthaceae	*B (2017 TCM-C)*	Unlisted				5
*Cydonia oblonga* Mill.	Rosaceae	*A (in lit.)*	Unlisted	E			
*Cymbopogon citratus* (DC.) Stapf	Poaceae	*B (2017 market)*	Unlisted			1	
*Cynodon aethiopicus* Clayton & J.R.Harlan	Poaceae	*A (in lit.)*	Unlisted				
*Cynomorium coccineum* L.	Cynomoriaceae	*B (2017 TCM-C)*	Unlisted				1
*Cyperus esculentus* L.	Cyperaceae	*A (in lit.)*	Unlisted				
*Cyperus rotundus* L.	Cyperaceae	*B (2017 TCM-C)*	Unlisted				1
*Cytisus scoparius* (L.) Link	Fabaceae	*A (in lit.)*	1a	E			
*Dactylis glomerata* L.	Poaceae	*A (in lit.)*	Unlisted	introduced			
*Datura ferox* L.	Solanaceae	*A (in lit.)*	1b	E			
*Datura metel* L.	Solanaceae	*A (market & in lit.)*	Unlisted	E			
*Datura* sp.	Solanaceae	*A (in lit.)*					
*Datura stramonium* L.	Solanaceae	*A (market & in lit.)*	1b	E			
*Datura wrightii* Regel	Solanaceae	*A (in lit.)*	1b				
*Daucus carota* L.	Apiaceae	*A (in lit.)*	Unlisted				
*Dianthus caryophyllus* L.	Caryophyllaceae	*B (2017 TCM-C)*	Unlisted				1
*Dianthus chinensis* L.	Caryophyllaceae	*B (2017 TCM-C)*	Unlisted				1
*Digitaria sanguinalis* (L.) Scop.	Poaceae	*A (in lit.)*	Unlisted				
*Dimocarpus longan* Lour.	Sapindaceae	*B (2017 TCM-C)*	Unlisted				1
*Dioscorea oppositifolia* L.	Dioscoreaceae	*B (2017 TCM-C)*	Unlisted				7
*Dracaena angolensis* (Welw. ex Carrière) Byng & Christenh.	Dracaenaceae	*A (2011 market)*	Unlisted		3		
*Dracaena fasciata* (Cornu ex Gérôme & Labroy) Byng & Christenh	Dracaenaceae	*A (2011 market)*	Unlisted		1		
*Duchesnea indica* (Jacks.) Focke	Rosaceae	*A (in lit.)*	1b	E			
*Duranta erecta* L.	Verbenaceae	*A (in lit.)*	3 GP, KZN, LP, MP, NW	E			
*Dysphania ambrosioides* (L.) Mosyakin & Clemants	Chenopodiaceae	*A (market & in lit.)*	Unlisted				
*Echinochloa frumentacea* Link	Poaceae	*B (2017 market)*	Unlisted			2	
*Eclipta prostrata* (L.) L.	Asteraceae	*A (market & in lit.); B (2017 TCM-C)*	Unlisted				1
*Elettaria cardamomum* (L.) Maton	Zingiberaceae	*B (2017 market)*	Unlisted			5	
*Eleutherine bulbosa* (Mill.) Urb.	Iridaceae	*A (2011 market)*	Unlisted		4		
*Elytrigia repens* (L.) Gould	Poaceae	*A (in lit.)*	Unlisted				
*Ephedra* sp.	Ephedraceae	*B (2017 TCM-C)*					4
*Epimedium* sp.	Berberidaceae	*B (2017 TCM-C)*					1
*Erigeron canadensis* L.	Asteraceae	*A (in lit.)*	Unlisted				
*Erigeron sumatrensis* Retz.	Asteraceae	*A (in lit.)*	Unlisted				
*Eriobotrya japonica* (Thunb.) Lindl.	Rosaceae	*A (market & in lit.); B (2017 TCM-C)*	1b WC and forest biome	E			1
*Erodium cicutarium* (L.) L'Hér.	Geraniaceae	*A (in lit.)*	Unlisted				
*Erythrostemon gilliesii* (Hook.) Klotzch	Fabaceae	*A (in lit.)*	1b	E			
*Eschscholzia californica* subsp. californica	Papaveraceae	*A (in lit.)*	Unlisted	C2			
*Ethulia conyzoides* L.f.	Asteraceae	*A (in lit.)*	Unlisted				
*Eucalyptus camaldulensis* Dehnh.	Myrtaceae	*A (in lit.)*	1b and 2	E			
*Eucalyptus globulus* Labill.	Myrtaceae	*A (in lit.)*	Unlisted	E			
*Eucalyptus grandis* W.Hill ex Maiden	Myrtaceae	*A (in lit.)*	1b and 2	E			
*Eucalyptus* sp.	Myrtaceae	*A (market & in lit.)*					
*Eucommia ulmoides* Oliv.	Eucommiaceae	*B (2017 TCM-C)*	Unlisted				2
*Euphorbia helioscopia* L.	Euphorbiaceae	*A (in lit.)*	Unlisted				
*Euphorbia heterophylla* L.	Euphorbiaceae	*A (in lit.)*	Unlisted	E			
*Euphorbia indica* Lam.	Euphorbiaceae	*A (in lit.)*	Unlisted	E			
*Euphorbia lathyris* L.	Euphorbiaceae	*A (in lit.)*	Unlisted	E			
*Euphorbia peplus* L.	Euphorbiaceae	*A (in lit.)*	Unlisted	C2			
*Euphorbia prostrata* Aiton **^CAII^**	Euphorbiaceae	*A (in lit.)*	Unlisted				
*Euphorbia tithymaloides* L.	Euphorbiaceae	*A (2011 market)*	Unlisted		5		
*Fagopyrum esculentum* Moench	Polygonaceae	*A (in lit.)*	Unlisted				
*Foeniculum vulgare* Mill.	Apiaceae	*A (2011 market); B (2017 market); B (2017 TCM-C)*	Unlisted	E	12	3	2
*Forsythia suspensa* (Thunb.) Vahl	Oleaceae	*B (2017 TCM-C)*	Unlisted				3
*Fragaria vesca* L.	Rosaceae	*A (in lit.)*	Unlisted				
*Fritillaria cirrhosa* D.Don	Liliaceae	*B (2017 TCM-C)*	Unlisted				2
*Fumaria muralis* Sond. ex W.D.J.Koch	Fumariaceae	*A (in lit.)*	Unlisted	C2			
*Galinsoga parviflora* Cav.	Asteraceae	*A (in lit.)*	Unlisted	E			
*Ganoderma lucidum* (Curtis) P. Karst	Ganodermataceae	*B (2017 TCM-C)*	Unlisted				2
*Garcinia kola* Heckel	Clusiaceae	*B (2017 market)*	Unlisted			3	
*Gardenia jasminoides* J.Ellis	Rubiaceae	*B (2017 TCM-C)*	Unlisted				5
*Gastrodia elata* Blume **^CAII^**	Orchidaceae	*B (2017 TCM-C)*	Unlisted				3
*Gentiana crassicaulis* Duthie ex Burkill	Gentianaceae	*B (2017 TCM-C)*	Unlisted				1
*Gentiana manshurica* Kitag	Gentianaceae	*B (2017 TCM-C)*	Unlisted				1
*Ginkgo biloba* L.	Ginkgoaceae	*B (2017 TCM-C)*	Unlisted				1
*Gladiolus* sp.	Iridaceae	*B (2017 market)*				3	
*Glebionis segetum* (L.) Fourr.	Asteraceae	*A (in lit.)*	Unlisted				
*Gleditsia triacanthos* L.	Fabaceae	*A (in lit.)*	1b excluding sterile cultivars	E			
*Glycyrrhiza glabra* L.	Fabaceae	*A (2011 market); B (2017 market); B (2017 TCM-C)*	Unlisted		20	2	37
*Glyphaea brevis* (Spreng.) Monach	Malvaceae	*B (2017 market)*	Unlisted			2	
*Gnetum africanum* Welw.	Gnetaceae	*B (2017 market)*	Unlisted			1	
*Gomphrena celosioides* Mart.	Amaranthaceae	*A (in lit.)*	Unlisted	E			
*Gomphrena globosa* L.	Amaranthaceae	*A (in lit.)*	Unlisted				
*Gymnanthemum amygdalinum* (Delile) Sch.Bip.	Asteraceae	*B (2017 market)*	Unlisted			3	
*Gynostma pentaphyllum* (Thunb.) Makino	Cucurbitaceae	*B (2017 TCM-C)*	Unlisted				1
*Hansenia weberbaueriana* (Fedde ex H.Wolff) Pimenov & Kljuykov	Apiaceae	*B (2017 TCM-C)*	Unlisted				3
*Hedychium gardnerianum* Sheppard ex Ker Gawl.	Zingiberaceae	*A (2011 market)*	1b	E	11		
*Heinsia crinita* (Wennberg) G.Taylor	Rubiaceae	*B (2017 market)*	Unlisted			5	
*Helianthus annuus* L.	Asteraceae	*A (in lit.)*	Unlisted	E			
*Helicteres isora* L.	Malvaceae	*A (2011 market)*	Unlisted		21		
*Helminthotheca echioides* (L.) Holub	Asteraceae	*A (in lit.)*	Unlisted				
*Hibiscus sabdariffa* L.	Malvaceae	*B (2017 market)*	Unlisted			1	
*Hordeum murinum* L.	Poaceae	*A (in lit.)*	Unlisted	E			
*Houttuynia cordata* Thunb.	Saururaceae	*B (2017 TCM-C)*	3	NA			2
*Hypericum perforatum* L.	Hypericaceae	*A (in lit.)*	2	E			
*Ibicella lutea* (Lindl.) Van Eselt.	Pedaliaceae	*A (market & in lit.)*	Unlisted				
*Illicium verum* Hook.f.	Schisandraceae	*B (2017 market)*	Unlisted			3	
*Impatiens walleriana* Hook.f.	Balsaminaceae	*A (in lit.)*	Unlisted				
*Ipomoea alba* L.	Convolvulaceae	*A (market & in lit.)*	1b	E			
*Ipomoea batatas* (L.) Lam.	Convolvulaceae	*A (in lit.)*	Unlisted				
*Ipomoea indica* (Burm.f.) Merr. Var.	Convolvulaceae	*A (in lit.)*	1b	E			
*Ipomoea purpurea* (L.) Roth	Convolvulaceae	*A (in lit.)*	1b	E			
*Iris domestica* (L.) Goldblatt & Mabb.	Iridaceae	*A (in lit.)*	Unlisted				
*Jasminum* sp.	Oleaceae	*B (2017 TCM-C)*					1
*Jatropha curcas* L.	Euphorbiaceae	*A (in lit.)*	2	E			
*Jatropha podagrica* Hook.	Euphorbiaceae	*A (market & in lit.)*	Unlisted				
*Juncus effusus* L.	Juncaceae	*B (2017 TCM-C)*	Unlisted	D1			1
*Kalanchoe delagoensis* Eckl. & Zeyh.	Crassulaceae	*A (2011 market)*	1b	E	1		
*Kalanchoe pinnata* (Lam.) Pers.	Crassulaceae	*A (market & in lit.)*	1b	E			
*Lablab purpureus* (L.) Sweet	Fabaceae	*A (in lit.)*	Unlisted				
*Lactuca serriola* L.	Asteraceae	*A (in lit.)*	Unlisted	E			
Laminariaceae sp.	Laminariaceae	*B (2017 TCM-C)*					1
*Lantana camara* L.	Fabaceae	*A (in lit.)*	1b	E			
*Lavandula angustifolia* Mill.	Lamiaceae	*B (2017 TCM-C)*	Unlisted				1
*Lepidium sativum* L.	Brassicaceae	*B (2017 market)*	Unlisted			6	
*Leucanthemum vulgare* (Vaill.) Lam.	Asteraceae	*A (in lit.)*	Unlisted	E			
*Ligusticum striatum* DC.	Apiaceae	*B (2017 TCM-C)*	Unlisted				19
*Ligustrum* sp.	Oleaceae	*B (2017 TCM-C)*					1
*Lilium lancifolium* Thunb.	Liliaceae	*B (2017 TCM-C)*	Unlisted				1
*Linum usitatissimum* L.	Linaceae	*B (2017 market)*	Unlisted			7	
*Lippia abyssinica* (Otto & A.Dietr.) Cufod.	Verbenaceae	*B (2017 market)*	Unlisted			2	
*Lolium perenne* L.	Poaceae	*A (in lit.)*	Unlisted	introduced			
*Lolium temulentum* L.	Poaceae	*A (in lit.)*	Unlisted				
*Lonicera confusa* DC.	Caprifoliaceae	*B (2017 TCM-C)*	Unlisted				5
*Lycium barbarum* L.	Solanaceae	*B (2017 TCM-C)*	Unlisted				1
*Macadamia* sp.	Proteaceae	*A (2011 market)*			1		
*Magnolia biondii* Pamp.	Magnoliaceae	*B (2017 TCM-C)*	Unlisted				1
*Magnolia officinalis* Rehder & E.H.Wilson	Magnoliaceae	*B (2017 TCM-C)*	Unlisted				7
*Malva parviflora* L.	Malvaceae	*A (market & in lit.)*	Unlisted	E			
*Malva pusilla* Sm.	Malvaceae	*A (in lit.)*	Unlisted				
*Malva verticillata* L.	Malvaceae	*A (market & in lit.)*	1b	E			
*Mangifera indica* L.	Anacardiaceae	*A (in lit.)*	Unlisted	E			
*Manihot esculenta* Crantz	Euphorbiaceae	*A (in lit.)*	Unlisted	E			
*Marrubium vulgare* L.	Lamiaceae	*A (in lit.)*	Unlisted				
*Melaleuca leucadendra* (L.) L.	Myrtaceae	*A (2011 market)*	Unlisted		12		
*Melia azedarach* L.	Meliaceae	*A (in lit.)*	1b and 3 in urban areas	E			
*Melilotus indicus* (L.) All.	Fabaceae	*A (in lit.)*	Unlisted	E			
*Melilotus officinalis* (L.) Lam.	Fabaceae	*A (in lit.)*	Unlisted				
*Mentha canadensis* L.	Lamiaceae	*B (2017 TCM-C)*	Unlisted				5
*Mentha spicata* L.	Lamiaceae	*A (in lit.)*	Unlisted				
*Mercurialis annua* L.	Euphorbiaceae	*A (in lit.)*	Unlisted				
*Mesosphaerum pectinatum* (L.) Kuntze	Lamiaceae	*A (in lit.)*	Unlisted	A0			
*Mimosa pigra* L.	Fabaceae	*A (market & in lit.)*	1b	E			
*Mimosa pudica* var. *hispida* Brenan	Fabaceae	*A (2011 market)*	Unlisted	E	1		
*Mirabilis jalapa* L.	Nyctaginaceae	*A (in lit.)*	1b	E			
*Momordica charantia* L.	Cucurbitaceae	*A (in lit.); B (2017 market)*	Unlisted	C2		1	
*Monodora myristica* (Gaertn.) Dunal	Annonaceae	*B (2017 market)*	Unlisted			3	
*Morinda morindoides* (Baker) Milne-Redh.	Rubiaceae	*B (2017 market)*	Unlisted			1	
*Moringa oleifera* Lam.	Moringaceae	*B (2017 market)*	Unlisted	E		6	
*Morus alba* L.	Moraceae	*B (2017 TCM-C)*	3	E			2
*Musa* x *paradisiaca* L.	Musaceae	*A (in lit.)*	Unlisted				
*Myristica fragrans* Houtt.	Myristicaceae	*B (2017 market)*	Unlisted			7	
*Myrtus communis* L.	Myrtaceae	*A (in lit.)*	Unlisted				
*Nasturtium officinale* W.T.Aiton	Brassicaceae	*A (in lit.)*	2	E			
*Nelumbo nucifera* Gaertn.	Nelumbonaceae	*B (2017 TCM-C)*	Unlisted				3
*Neolitsea cassia* (L.) Kosterm	Lauraceae	*B (2017 TCM-C)*	Unlisted				14
*Nepeta tenuifolia* Benth.	Lamiaceae	*B (2017 TCM-C)*	Unlisted				2
*Nerium oleander* L.	Apocynaceae	*A (market & in lit.)*	1b excluding sterile cultivars	E			
*Nicandra physalodes* (L.) Gaertn.	Solanaceae	*A (in lit.)*	1b	E			
*Nicotiana glauca* Graham	Solanaceae	*A (in lit.)*	1b	E			
*Nicotiana tabacum* L.	Solanaceae	*A (in lit.)*	Unlisted	E			
*Nigella sativa* L.	Ranunculaceae	*B (2017 market)*	Unlisted			13	
*Ocimum basilicum* L.	Lamiaceae	*B (2017 market)*	Unlisted			3	
*Ocimum gratissimum* L.	Lamiaceae	*B (2017 market)*	Unlisted			1	
*Oldenlandia affinis* (Roem. & Schult.) DC.	Rubiaceae	*B (2017 market)*	Unlisted			1	
*Ophiopogon japonicus* (Thunb.) Ker Gawl.	Asparagaceae	*B (2017 TCM-C)*	Unlisted				7
*Opuntia ficus-indica* (L.) Mill.	Cactaceae	*A (2011 market)*	1b excluding spineless cultivars	E	8		
*Opuntia monacantha* (Willd.) Haw.	Cactaceae	*A (in lit.)*	1b	E			
*Opuntia robusta* H.L.Wendl. ex Pfeiff.	Cactaceae	*A (2011 market)*	1a excluding spineless cultivars	E	6		
*Opuntia* sp.	Cactaceae	*A (market & in lit.)*					
*Oxalis corniculata* L.	Oxalidaceae	*A (in lit.)*	Unlisted	E			
*Paeonia anomala* L.	Paeoniaceae	*B (2017 TCM-C)*	Unlisted				5
*Paeonia lactiflora* Pall.	Paeoniaceae	*B (2017 TCM-C)*	Unlisted				20
*Paeonia suffruticosa* Andrews	Paeoniaceae	*B (2017 TCM-C)*	Unlisted				10
*Panax ginseng* C.A.Mey. **^CAII^**	Araliaceae	*B (2017 TCM-C)*	Unlisted				23
*Panax notoginseng* (Burkill) F.H.Chen	Araliaceae	*B (2017 TCM-C)*	Unlisted				3
*Paraserianthes lophantha* (Willd.) I.C.Nielsen subsp. *lophantha*	Fabaceae	*A (in lit.)*	1b	E			
*Parkinsonia aculeata* L.	Fabaceae	*A (in lit.)*	1b	E			
*Paspalum dilatatum* Poir.	Poaceae	*A (in lit.)*	Unlisted	E			
*Passiflora caerulea* L.	Passifloraceae	*A (in lit.)*	1b	E			
*Passiflora edulis* Sims.	Passifloraceae	*A (in lit.)*	2 GP, NW, KZN, LP, MP, EC	E			
*Passiflora suberosa* L.	Passifloraceae	*A (market & in lit.)*	1b	E			
*Perilla frutescens* (L.) Britton	Lamiaceae	*B (2017 TCM-C)*	Unlisted				1
*Persea americana* Mill.	Periplocaceae	*A (market & in lit.); B (2017 market)*	Unlisted	E		1	
*Persicaria hydropiper* (L.) Delarbre	Polygonaceae	*A (in lit.)*	Unlisted	E			
*Persicaria lapathifolia* (L.) Delarbre	Polygonaceae	*A (in lit.)*	Unlisted	E			
*Petroselinum crispum* (Mill.) Fuss	Apiaceae	*A (in lit.)*	Unlisted				
*Phellodendron amurense* Rupr.	Rutaceae	*B (2017 TCM-C)*	Unlisted				8
*Phoenix dactylifera* L.	Arecaceae	*B (2017 market)*	Unlisted	E		7	
*Phyllanthus emblica* L.	Phyllanthaceae	*B (2017 market)*	Unlisted			1	
*Physalis angulata* L.	Solanaceae	*A (in lit.)*	Unlisted	C2			
*Physalis peruviana* L.	Solanaceae	*A (in lit.)*	Unlisted	E			
*Phytolacca americana* L.	Phytolaccaceae	*A (in lit.)*	1b	E			
*Phytolacca dioica* L.	Phytolaccaceae	*A (market & in lit.)*	3	E			
*Phytolacca octandra* L.	Phytolaccaceae	*A (in lit.)*	1b	E			
*Pilea microphylla* (L.) Liebm.	Urticaceae	*A (market & in lit.)*	Unlisted				
*Pinellia ternata* (Thunb.) Makino	Araceae	*B (2017 TCM-C)*	Unlisted				9
*Pinus roxburghii* Sarg.	Pinaceae	*A (in lit.)*	2	E			
*Pinus* sp.	Pinaceae	*A (market & in lit.)*					
*Piper longum* L.	Piperaceae	*B (2017 market)*	Unlisted			1	
*Piper nigrum* L.	Piperaceae	*B (2017 market)*	Unlisted			10	
*Plantago asiatica* L.	Plantaginaceae	*B (2017 TCM-C)*	Unlisted				2
*Plantago lanceolata* L.	Plantaginaceae	*A (in lit.)*	Unlisted	E			
*Plantago major* L.	Plantaginaceae	*A (in lit.)*	Unlisted	E			
*Platycladus orientalis* (L.) Franco	Cupressaceae	*B (2017 TCM-C)*	Unlisted				2
*Platycodon grandiflorus* (Jacq.) A.DC.	Campanulaceae	*B (2017 TCM-C)*	Unlisted				8
*Plectranthus barbatus* Andrews.	Lamiaceae	*A (in lit.)*	1b	E			
*Plumbago zeylanica* L.	Plumbaginaceae	*A (in lit.)*	Unlisted				
*Poa annua* L.	Poaceae	*A (in lit.)*	Unlisted	E			
*Pogoston cablin* (Blanco) Benth	Lamiaceae	*B (2017 TCM-C)*	Unlisted				1
*Polycarpaea corymbosa* (L.) Lam. var. *corymbosa*	Caryophyllaceae	*A (in lit.)*	Unlisted				
*Polygala* sp.	Polygalaceae	*B (2017 TCM-C)*					3
*Polygonum aviculare* L.	Polygonaceae	*A (in lit.); B (2017 TCM-C)*	Unlisted	C2			1
*Polyporus umbellatus* (Pers.) Fr.	Polyporaceae	*B (2017 TCM-C)*	Unlisted				2
*Populus* x *canescens* (Aiton) Sm.	Salicaceae	*A (in lit.)*	2				
*Portulaca oleracea* L.	Portulacaceae	*A (in lit.)*	Unlisted	E			
*Proboscidea louisianica* (Mill.) Thell. subsp.	Martyniaceae	*A (in lit.)*	Unlisted				
*Proboscidea louisianica* (Mill.) Thell. subsp. *fragrans* (Lindl.)	Martyniaceae	*A (market & in lit.)*	Unlisted				
*Prunella vulgaris* L.	Lamiaceae	*A (in lit.)*	Unlisted				
*Prunus armeniaca* L.	Rosaceae	*B (2017 TCM-C)*	Unlisted	E			5
*Prunus davidiana* (Carrière) Franch.	Rosaceae	*B (2017 TCM-C)*	Unlisted				5
*Prunus persica* var. *persica*	Rosaceae	*A (in lit.)*	Unlisted	E			
*Psidium guajava* L.	Myrtaceae	*A (in lit.)*	2 and 3 (see NEM:BA)	E			
*Pteridium aquilinum* subsp. *aquilinum* (L.) Khum	Dennstaedtiaceae	*A (in lit.)*	Unlisted				
*Pterocarpus santalinus* L.f. **^CAII^**	Fabaceae	*B (2017 market)*	Unlisted			3	
*Pueraria montana* (Lour.) Merr.	Fabaceae	*B (2017 TCM-C)*	1a	E			2
*Punica granatum* L.	Punicaceae	*A (in lit.)*	Unlisted	E			
*Quassia africana* (Baill.) Baill.	Simaroubaceae	*B (2017 market)*	Unlisted			3	
*Quercus infectoria* G.Olivier	Fagaceae	*A (2011 market)*	Unlisted		2		
*Quercus robur* L.	Fagaceae	*A (in lit.)*	Unlisted	E			
*Raphanus raphanistrum* L. subsp.	Brassicaceae	*A (in lit.)*	Unlisted	E			
*Raphanus raphanistrum* subsp. *sativus* (L.) Domin	Brassicaceae	*A (in lit.)*	Unlisted				
*Rehmannia glutinosa* (Gaertn.) DC.	Plantaginaceae	*B (2017 TCM-C)*	Unlisted				19
*Reynoutria multiflora* (Thunb.) Moldenke	Polygonaceae	*B (2017 TCM-C)*	Unlisted				3
*Rheum palmatum* var. *tanguticum* Maxim. ex Balf.	Polygonaceae	*B (2017 TCM-C)*	Unlisted				9
*Richardia brasiliensis* Gomes	Rubiaceae	*A (in lit.)*	Unlisted	E			
*Ricinus communis* L.	Euphorbiaceae	*A (2011 market)*	2	E	6		
*Robinia pseudoacacia* L.	Fabaceae	*A (in lit.)*	1b	E			
*Rosa chinensis* Jacq.	Rosaceae	*B (2017 TCM-C)*	Unlisted				1
*Rosmarinus officinalis* var. L.	Lamiaceae	*A (in lit.); B (2017 market)*	Unlisted			3	
*Rumex acetosella* L.	Polygonaceae	*A (in lit.)*	1a Prince Edward & Marion Islands	C2			
*Rumex crispus* L.	Polygonaceae	*A (market & in lit.)*	Unlisted	E			
*Ruta chalepensis* Mill.	Rutaceae	*B (2017 market)*	Unlisted			2	
*Ruta graveolens* L.	Rutaceae	*A (in lit.)*	Unlisted				
*Salix babylonica* L.	Salicaceae	*A (in lit.)*	Unlisted	E			
*Salvadora persica* L.	Salvadoraceae	*B (2017 market)*	Unlisted			9	
*Salvia coccinea* Buc'hoz ex Etl.	Lamiaceae	*A (in lit.)*	Unlisted	E			
*Salvia hispanica* L.	Lamiaceae	*B (2017 market)*	Unlisted			2	
*Salvia miltiorrhiza* Bunge	Lamiaceae	*B (2017 TCM-C)*	Unlisted				7
*Salvia officinalis* L.	Lamiaceae	*A (in lit.)*	Unlisted				
*Sanguisorba officinalis* L.	Rosaceae	*B (2017 TCM-C)*	Unlisted				1
*Saposhnikovia divaricata* (Turcz. ex Ledeb) Schischk.	Apiaceae	*B (2017 TCM-C)*	Unlisted				6
*Schinus molle* L.	Dracaenaceae	*A (in lit.)*	Unlisted	E			
*Schisandra* sp.	Magnoliaceae	*B (2017 TCM-C)*					10
*Schkuhria pinnata* (Lam.) Kuntze ex Thell.	Asteraceae	*A (in lit.)*	Unlisted	E			
*Schlumbergera truncata* (Haw.) Moran **^CAII^**	Cactaceae	*A (market & in lit.)*	Unlisted				
*Scrophularia ningpoensis* Hemsl.	Scrophulariaceae	*B (2017 TCM-C)*	Unlisted				5
*Scutellaria baicalensis* Georgi	Lamiaceae	*B (2017 TCM-C)*	Unlisted				17
*Senecio vulgaris* L.	Asteraceae	*A (in lit.)*	Unlisted				
*Senna alexandrina* Mill.	Fabaceae	*A (2011 market)*	Unlisted		4		
*Senna didymobotrya* (Fresen.) H.S.Irwin & Barneby	Fabaceae	*A (market & in lit.)*	1b EC, WC, KZN, LP, MP	E			
*Senna occidentalis* (L.) Link	Fabaceae	*A (in lit.)*	1b	E			
*Senna septemtrionalis* (Viv.) H.S.Irwin & Barneby	Fabaceae	*A (in lit.)*	1b	E			
*Senna* sp.	Fabaceae	*A (2011 market); B (2017 TCM-C)*			1		1
*Sesamum indicum* L.	Pedaliaceae	*A (in lit.); B (2017 TCM-C)*	Unlisted				1
*Setaria italica* (L.) P.Beauv.	Poaceae	*A (in lit.)*	Unlisted				
*Sigesbeckia orientalis* L.	Asteraceae	*A (in lit.)*	Unlisted	E			
*Silybum marianum* (L.) Gaertn.	Asteraceae	*A (in lit.)*	Unlisted	E			
*Sinapis alba* L.	Brassicaceae	*A (in lit.)*	Unlisted				
*Smilax glabra* Roxb.	Smilacaceae	*B (2017 TCM-C)*	Unlisted				1
*Solanum americanum* Mill.	Solanaceae	*A (in lit.)*	Unlisted				
*Solanum mauritianum* Scop.	Solanaceae	*A (in lit.)*	1b	E			
*Solanum pseudocapsicum* L.	Solanaceae	*A (in lit.)*	1b	E			
*Solanum rostratum* Dunal.	Solanaceae	*A (in lit.)*	Unlisted	C2			
*Sonchus oleraceus* (L.) L.	Asteraceae	*A (in lit.)*	Unlisted	E			
*Sorghum halepense* (L.) Pers	Poaceae	*A (in lit.)*	2	E			
*Sparaxis grandiflora* (D.Delaroche) Ker Gawl.	Iridaceae	*A (in lit.)*	Unlisted				
*Spergula arvensis* L.	Caryophyllaceae	*A (in lit.)*	Unlisted				
*Stellaria media* (L.) Vill.	Caryophyllaceae	*A (in lit.)*	1a Prince Edward; 1b Marion Island	C2			
*Stemona* sp.	Stemonaceae	*B (2017 TCM-C)*					2
*Stephania tetrandra* S.Moore	Menispermaceae	*B (2017 TCM-C)*	Unlisted				2
*Styphnolobium japonicum* (L.) Schott	Fabaceae	*A (in lit.)*	Unlisted	E			
*Tagetes minuta* L.	Asteraceae	*A (in lit.)*	Unlisted	E			
*Tamarindus indica* L.	Fabaceae	*A (in lit.)*	Unlisted				
*Taraxacum campylodes* G.E.Haglund	Asteraceae	*A (in lit.)*	Unlisted				
*Taraxacum mongolicum* Hand. -Mazz.	Asteraceae	*B (2017 TCM-C)*	Unlisted				1
*Taxillus chinensis* (DC.) Danser	Loranthaceae	*B (2017 TCM-C)*	Unlisted				1
*Tephrosia densiflora* Hook. f.	Fabaceae	*A (in lit.)*	Unlisted				
*Tephrosia purpurea* (L.) Pers.	Fabaceae	*A (in lit.)*	Unlisted				
*Terminalia chebula* Retz.	Combretaceae	*A (2011 market)*	Unlisted		4		
*Tetrapanax papyrifer* (Hook.) K.Koch	Araliaceae	*B (2017 TCM-C)*	Unlisted				1
*Theobroma cacao* L.	Malvaceae	*B (2017 market)*	Unlisted			1	
*Thymus schimperi* Ronniger	Lamiaceae	*B (2017 market)*	Unlisted			2	
*Tillandsia fasciculata* Sw.	Bromeliaceae	*A (in lit.)*	Unlisted				
*Tithonia diversifolia* (Hemsl.) A.Gray	Asteraceae	*A (in lit.)*	1b	E			
*Trachyspermum ammi* (L.) Sprague	Apiaceae	*B (2017 market)*	Unlisted			4	
*Tragopogon porrifolius* L.	Asteraceae	*A (in lit.)*	Unlisted				
*Tribulus terrestris* L.	Zygophyllaceae	*B (2017 TCM-C)*	Unlisted				2
*Tridax procumbens* L.	Asteraceae	*A (in lit.)*	Unlisted	E			
*Trifolium pratense* L.	Fabaceae	*A (in lit.)*	Unlisted				
*Trigonella foenum-graecum* L.	Fabaceae	*B (2017 market)*	Unlisted			7	
*Tussilago farfara* L.	Asteraceae	*B (2017 TCM-C)*	Unlisted				1
*Typha* sp.	Typhaceae	*B (2017 TCM-C)*					1
*Uncaria sinensis* (Oliv.) Havil.	Rubiaceae	*B (2017 TCM-C)*	Unlisted				2
*Urtica dioica* L.	Urticaceae	*A (in lit.)*	Unlisted				
*Urtica urens* L.	Urticaceae	*A (in lit.)*	Unlisted				
*Verbena officinalis* L.	Verbenaceae	*A (in lit.)*	Unlisted	E			
*Verbena rigida* Spreng	Verbenaceae	*A (in lit.)*	1b	E			
*Vernicia fordii* (Hemsl.) Airy Shaw	Euphorbiaceae	*A (in lit.)*	Unlisted				
*Vinca major* L.	Apocynaceae	*A (in lit.)*	1b	E			
*Vincetoxicum glaucescens* (Decne.) C.Y.Wu & D.Z.Li	Apocynaceae	*B (2017 TCM-C)*	Unlisted				1
*Viola tricolor* L.	Violaceae	*A (in lit.)*	Unlisted				
*Vitellaria paradoxa* C.F.Gaertn.	Sapotaceae	*B (2017 market)*	Unlisted			1	
*Withania somnifera* (L.) Dunal	Solanaceae	*A (2011 market)*	Unlisted		14		
*Wolfiporia extensa* (Peck) Ginns	Polyporaceae	*B (2017 TCM-C)*	Unlisted				
*Wurfbainia longiligularis* (T.L.Wu) Skornick. & A.D.Poulsen	Zingiberaceae	*B (2017 TCM-C)*	Unlisted				1
*Wurfbainia villosa* (Lour.) Skornick. & A.D.Poulsen	Zingiberaceae	*B (2017 TCM-C)*	Unlisted				1
*Xanthium spinosum* L.	Asteraceae	*A (in lit.)*	1b	E			
*Xanthium strumarium* L.	Asteraceae	*A (in lit.); B (2017 TCM-C)*	1b	E			
*Xylopia aethiopica* (Dunal) A.Rich.	Annonaceae	*B (2017 market)*	Unlisted			1	
*Zingiber officinale* Roscoe	Zingiberaceae	*A (in lit.); B (2017 market); B (2017 TCM-C)*	Unlisted			16	14
*Ziziphus jujuba* Mill.	Rhamnaceae	*B (2017 market)*	Unlisted			3	1

**Fig. 1:**
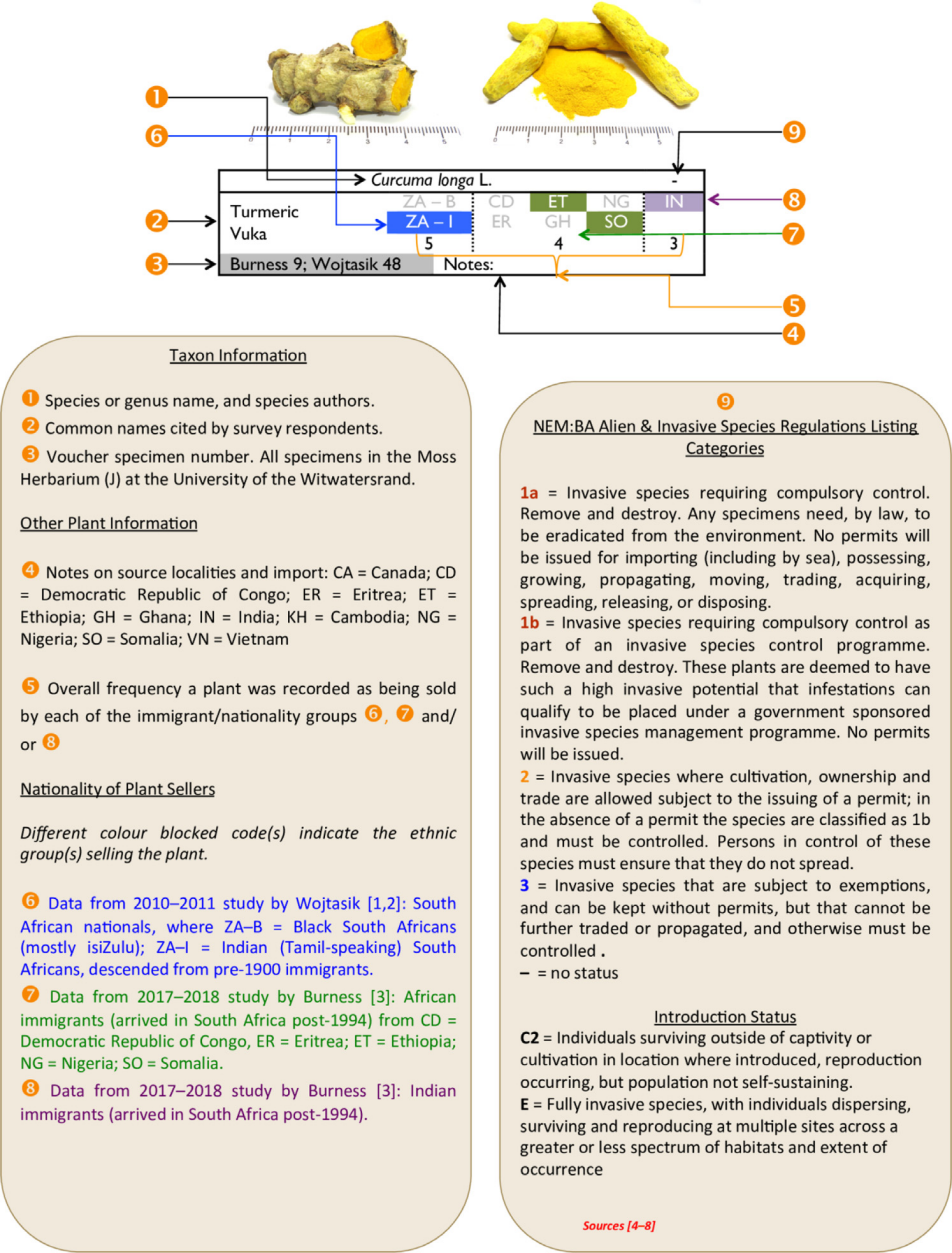
Key to the photographic guides in Tables 1 and 2.

Regarding the photographic guide for 96 alien plants (Tables 1 and 2): accompanying each photograph of the specimens is the following information: species name; common name(s) provided by the survey respondents; language of common name; voucher specimen numbers in the Moss Herbarium (J) of the University of the Witwatersrand; invasive alien plant category (according to NEM:BA A&IS regulations [4,5]); nationality of the medicine traders; and, notes on source localities (e.g. imported or collected in southern Africa). A key explaining the photographic guide is given in Fig. 1.

**Fig. 2. a–e:**
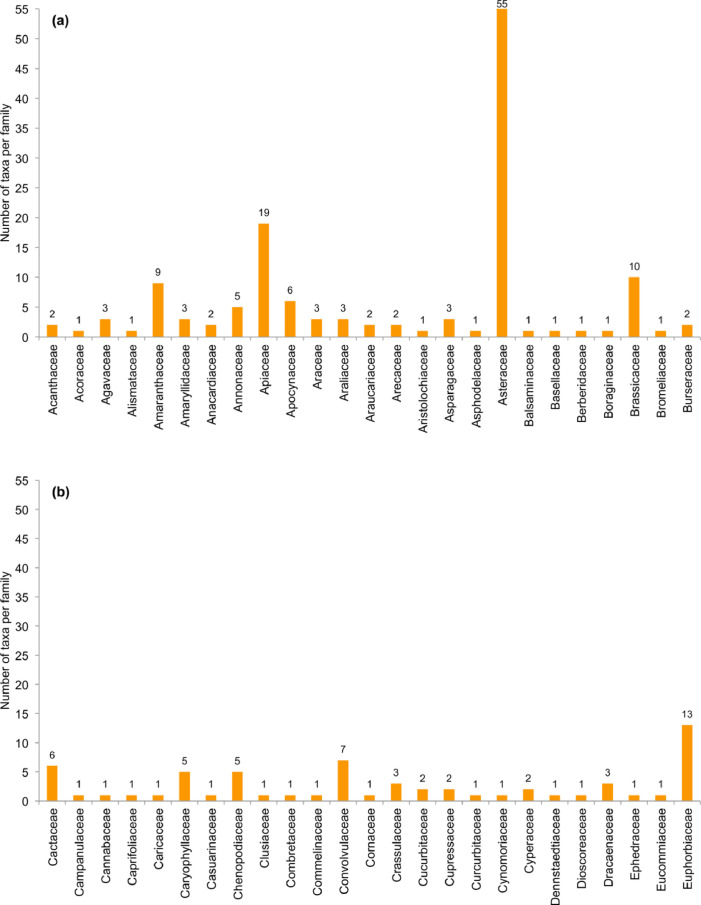

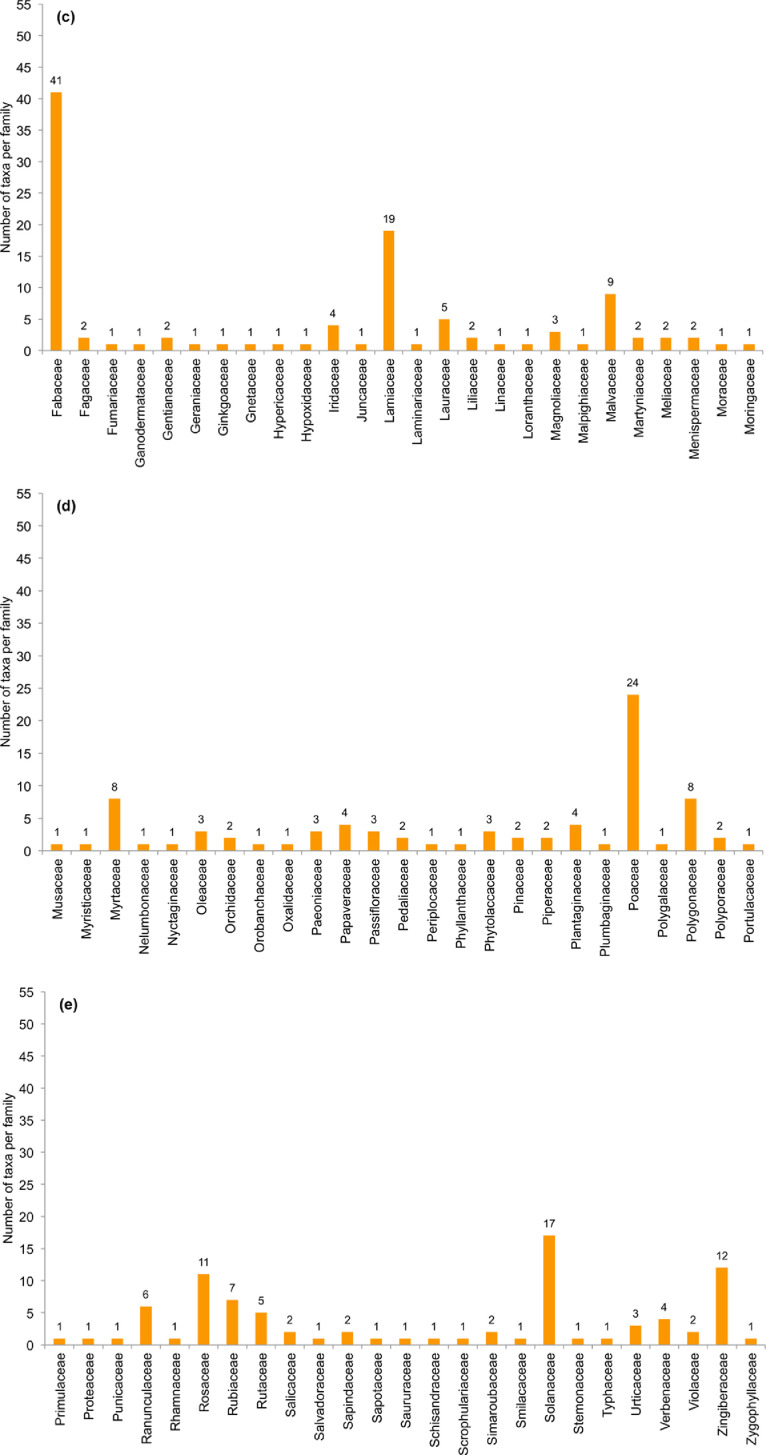
Number of alien taxa per family listed in Table 4. Families are presented alphabetically and spread over five figures: (**a**) family names starting with A to B, (**b**) names starting with C to E, (**c**) names starting with F to Mor, (**d**) names starting with Mus to Por, and (**e**) names starting with Pri to Z.

Regarding the species inventory for 475 alien plants (Table 3): the species were documented in two separate studies (designated by A and B, and conducted in 2010/2011 and 2017/2018 respectively in Table 3). In study A, the information originated from (i) a survey of traders in healthcare outlets/markets in Johannesburg, Pretoria and Durban in 2010/2011 *(2011 market*); and (ii) a literature survey, where species are classified according to whether they were recorded as being sold in a market or not (respectively, *market & in lit.* and *in lit.*) [1]. In study B, the information originated from (i) a survey of Indian, and West and East African traders in markets in Johannesburg in *(2017 market)*; and (ii) from a catalogue of remedies sold in at least four TCM franchises in Johannesburg *(2017 TCM-C.)* [3]. The numbers of alien taxa per family for the consolidated data sets are shown in Fig. 2. The number of taxa that were identified in each study and information type (e.g. A (2011 market) or B (2017 TCM-C)) are in Table 4. Furthermore, the species that were documented in more than one study are listed next to the information source. Information on the species includes their legal and introduction status in South Africa (as per [4,8]), and the number of taxa in these categories is summarised in Tables 5 and 6.

**Table 4:** Combined number of taxa per information source category and survey, where: *A* = 2010/2011; *B* = 2017/2018; *market* = obtained from a survey of traders selling alien medicinal plants in markets or outlets such as shops in Johannesburg, Pretoria and/or Durban; *in lit.* = obtained from a literature survey of alien plants used in South Africa; *market & in lit.* = recorded in the literature as being sold in a market/outlet in South Africa; *TCM-C* = obtained from a catalogue of Traditional Chinese Medicine remedies sold in at least four franchises in Johannesburg. Species recorded from >1 different information sources are listed.

Study and information source	No. taxa	Species common to >1 information source
*A (2011 market)*	27	
*A (2011 market); B (2017 market)*	3	*Brassica* sp., *Coriandum sativum*, Curcuma *longa*
*A (2011 market); B (2017 market); B (2017 TCM-C)*	2	*Foeniculum vulgare, Glycyrrhiza glabra*
*A (2011 market); B (2017 TCM-C)*	4	*Acorus calamus, Areca catechu, Coix lacryma-jobi, Senna* sp.
*A (in lit.)*	223	
*A (in lit.); B (2017 market)*	3	*Allium sativum, Momordica charantia, Rosmarinus officinalis* var.
*A (in lit.); B (2017 market); B (2017 TCM-C)*	2	*Anethum graveolens, Zingiber officinale*
*A (in lit.); B (2017 TCM-C)*	3	*Polygonum aviculare, Xanthium strumarium*
*A (market & in lit.)*	28	
*A (market & in lit.); B (2017 market)*	2	*Annona reticulata, Persea americana*
*A (market & in lit.); B (2017 market); B (2017 TCM-C)*	1	*Cinnamomum verum*
*A (market & in lit.); B (2017 TCM-C)*	2	*Eclipta prostrata, Eriobotyra japonica*
*B (2017 market)*	55	
*B (2017 TCM-C)*	120	
Total plants	475*	

⁎ In addition to the 475 identified taxa, there were and 12 and 8 unidentified plants were recorded in studies A (2011 market) and B (2017 market) respectively.

**Table 5:** Number and percentage of alien plant species assigned a legal status category (according to NEM:BA A&IS regulations), where: category 1a = invasive species that are targets for national eradication; 1b = invasive species that must be controlled; 2 = invasive species where cultivation, ownership and trade are allowed subject to the issuing of a permit, and that must be controlled in the absence of a permit; 3 = invasive species that are subject to exemptions, but that cannot be further traded or propagated, and otherwise must be controlled; unlisted = alien species that are not listed in the regulations, but that have been reported as present outside of captivity or cultivation in South Africa or on offshore islands.

Legal status category	Number of species (S=447)*	Percentage of total taxa⁎⁎
1a	4	0.8%
1a & 1b	1	0.2%
1b	56	11.8 %
1b & 2	2	0.4%
1b & 3	3	0.6%
2	14	2.9%
3	5	1.0%
2 & 3	1	0.2%
Unlisted	361	76.0%

⁎ Excludes 28 taxa identified to genus only and taxa not identified to species level.

**Table 6:** Number and percentage of species assigned an Introduction status category in South Africa, where: A0 = never introduced beyond limits of indigenous range to South Africa; C0 = individuals released outside of captivity or cultivation in location where introduced, but incapable of surviving for a significant period; C2 = individuals surviving outside of captivity or cultivation in location where introduced, but population not self-sustaining; D1 = self-sustaining population outside of captivity or cultivation, with individuals surviving a significant distance from the original point of introduction; E = fully invasive species, with individuals dispersing, surviving and reproducing at multiple sites across a greater or less spectrum of habitats and extent of occurrence; introduced = introduced, but no other data available;

Introduction status category	Number of species (S=175)	Percentage of total taxa*
A0	1	0.2%
C0	1	0.2%
C2	18	3.8%
D1	1	0.2%
E	150	31.4%
Introduced	3	0.6%
NA	1	0.2%

⁎ a percentage of S = 475.

## 2 Experimental Design, Materials and Methods

Two semi-quantitative surveys of alien plant taxa were conducted. First, a survey of plants sold in the Faraday (Johannesburg) and Warwick (Durban) *umuthi* markets (n=32 and 28 traders respectively) was conducted from October 2010 and February 2011 [1,2]. In addition, 17 *umuthi* shops were visited in Johannesburg, Pretoria and Durban; 10 of these shops were owned by the descendants of Indian indentured labourers brought to South Africa from 1860 to work in the sugarcane plantations. Specimens were purchased during the survey visits and deposited as vouchers at the C.E. Moss Herbarium (J) at University of the Witwatersrand. The qualitative information recorded during the survey included: the vernacular name of the plant specimen, the botanical name of the plant (if known), the plant part type (e.g. seeds, tubers, bulbs, etc.), the means by which the plant was acquired (i.e. harvested or bought), and the geographic origin (Questionnaire in Appendix 1a). While specimens that could potentially regrow or germinate were tested for viability (e.g. seeds and tubers) [9], voucher specimens were not collected of the plants once they had emerged, flowered, or grown out sufficiently. Photographs of the voucher specimens were taken retrospectively in 2019.

Second, a survey of plants sold by immigrants and migrants from West and East Africa (*n*=25), India (*n*=4), and China (*n*=1), who arrived in South Africa after 1994, was conducted from November 2017 to April 2018 [3]. Specimens were purchased during the survey visits and deposited as vouchers at the C.E. Moss Herbarium (J) – however, the Chinese trader would not permit specimens to be purchased and consequently no photographic data on Chinese plants used in South Africa are included in the photographic guide. However, this part of the survey was based on plants listed in a catalogue that are allegedly imported from China [3]. The qualitative information recorded during the 2017/2018 survey included: the vernacular name of the plant specimen, the botanical name of the plant (if known), the plant part type (e.g. seeds, tubers, bulbs, etc.), the means by which the plant was acquired (i.e. harvested or bought), and the country of origin (Questionnaire in Appendix 1b). Specimens that could potentially regrow were tested for viability, and to identify them; voucher specimens were collected from these plants once they had emerged, flowered, or grown out sufficiently [3].

Plants are arranged alphabetically in the photo-guide according to genus and species (Tables 1 and 3), and unidentified plants for which photos were taken are in Table 2. Under every photograph is a table of information for the taxon obtained from the qualitative surveys, to which codes for the invasive alien plant category, and introduction status [4,8], were added. The numbers of traders from each nationality selling each species are also included. The online database *Plants of the World Online* [10] was used to cross-reference scientific names to find the currently accepted synonyms and plant families. The species in Table 3 were also checked against CITES (Convention on International Trade in Endangered Species of Wild Fauna and Flora) to establish whether international trade is regulated through import/export permits [11]. With the exception of information sourced from the literature, the frequencies (in Table 3) with which the species were recorded in the different surveys of traditional medicine outlets and the TCM catalogue are listed.

## Ethics Statement

The protocols for this research were approved in: (i) 2010 (Protocol H120509) [1], and (ii) 2017 (Protocol H17/07/02) [3] by the Human Research Ethics Committee (non-medical) of the University of the Witwatersrand. Informed consent was obtained from all respondents before commencing the research, and respondents could discontinue their participation at any time.

## CRediT authorship contribution statement

**Vivienne L. Williams:** Conceptualization, Methodology, Formal analysis, Writing – original draft, Supervision. **Amy Burness:** Methodology, Investigation, Data curation, Visualization. **Ewa M. Wojtasik:** Methodology, Investigation, Data curation. **Marcus J. Byrne:** Conceptualization, Methodology, Writing – original draft, Supervision.

## Declaration of Competing Interest

The authors declare that they have no known competing financial interests or personal relationships which have, or could be perceived to have, influenced the work reported in this article.

## Appendix Supplementary materials

Image, application 1
