# Supplementary material for: Dataset, including a photo-guide, of alien plants sold in traditional medicine markets and healthcare outlets in three South African cities, specifically by traders of Indian, West African, East African, and Chinese origin
Source: Data Brief. 2021 Sep 21;38:107395. doi: 10.1016/j.dib.2021.107395 (PMC8479245; doi:10.1016/j.dib.2021.107395)
Supplement: Supplementary file 1 [file mmc1.docx]

**Appendix 1a**

**Questionnaire used to gather invidual plant information in the 2010/2011 study by Wojtasik [1,2]**

| Trader code |  | Alien plant no |  | Plant part |  | | | Quantity (unit/%) |  |
| --- | --- | --- | --- | --- | --- | --- | --- | --- | --- |
| 1. Vernacular name 1. Botanical name | | | | | | | | | |
|  | | | | | |  |  | | |

1. Do you buy or harvest it or both?

| Buy it |  | Both |  | Harvest |  |
| --- | --- | --- | --- | --- | --- |

| 1. Origin: Where did you buy this plant? (E.g. Town, area, province). |  | 1. Origin: Where did you harvest this plant? (E.g. Town, area, province). |
| --- | --- | --- |
|  |  |  |

| 1. When did you buy this stock? (Time in weeks/ months since getting it?). |  | 1. When did you harvest this stock? (Time in weeks/ months since getting it?). |
| --- | --- | --- |
|  |  |  |

| 1. How much did you buy? (E.g. No. of bags and size of bags). |  | 1. How much did you harvest? (E.g. No. of bags and size of bags). |
| --- | --- | --- |
|  |  |  |

| 1. What quantity do you buy every month/year? | | | |  | 1. What quantity do you harvest every month/year? | | | |
| --- | --- | --- | --- | --- | --- | --- | --- | --- |
| Month |  | Year |  |  | Month |  | Year |  |

| 1. How do you prepare this plant before it is used? ( √ the correct box) | | | |  | 1. How do you prepare this plant before it is used?   ( √ the correct box) | | | |
| --- | --- | --- | --- | --- | --- | --- | --- | --- |
| Ground □ | Boiled  □ | Crushed □ | Scattered  □ |  | Ground  □ | Boiled  □ | Crushed □ | Scattered  □ |
| Other | | | |  | Other | | | |

| 1. Other info |  |  |
| --- | --- | --- |
|  | | |

**Appendix 1b**

**Questionnaire used to gather invidual plant information in the 2017/2018 study by Burness [3]**

Respondent information

| Trader code |  | Sex |  | | Ethnicity | |  | Date |  |
| --- | --- | --- | --- | --- | --- | --- | --- | --- | --- |
| Place Length of stay in South Africa | | | | | | | | | |
|  | | | |  | |  | | | |

Plant information

| Species # | Vernacular name | Plant part | Min cost/unit | Bupers/week | Stock/week | Where is it from? | How does it come to RSA | Uses |
| --- | --- | --- | --- | --- | --- | --- | --- | --- |
|  |  |  |  |  |  |  |  |  |
|  |  |  |  |  |  |  |  |  |
|  |  |  |  |  |  |  |  |  |
|  |  |  |  |  |  |  |  |  |
|  |  |  |  |  |  |  |  |  |
|  |  |  |  |  |  |  |  |  |
|  |  |  |  |  |  |  |  |  |
|  |  |  |  |  |  |  |  |  |
|  |  |  |  |  |  |  |  |  |
|  |  |  |  |  |  |  |  |  |
|  |  |  |  |  |  |  |  |  |
|  |  |  |  |  |  |  |  |  |

| Elaborate on how the plant comes to South Africa (what countries is stock dtransported through, and who imports the plant material) |
| --- |
|  |

| Notes: Observations |
| --- |
|  |
